# Examining the distinctiveness of body image and self-related constructs in eating disorders using virtual reality: the role of shape concerns, physical self-worth, and global self-worth

**DOI:** 10.3389/fpsyg.2025.1649698

**Published:** 2026-01-27

**Authors:** Johana Monthuy-Blanc, Gabrielle Fortin, Giulia Corno, Stéphane Bouchard

**Affiliations:** 1 Groupe de Recherche Loricorps, Université du Québec à Trois-Rivières, Trois-Rivières, QC, Canada; 2 Research Center of Mental Health University Institute of Montreal, Montreal (CR-IUSMM), Montréal, QC, Canada; 3 Centre intégré de santé et des services sociaux de l'Outaouais, Gatineau, QC, Canada; 4 Département de psychoéducation et psychologie, Université du Québec en Outaouais, Gatineau, QC, Canada

**Keywords:** body dissatisfaction, dysfunctional eating attitude and behaviors, eating concerns, eating pathology, physical self-perceptions, self-esteem, shape concerns, virtual reality

## Abstract

**Introduction:**

In Western culture, the female body is commonly socially perceived as an object of evaluation, causing women to frequently evaluate their self-worth based on their physical appearance. Since the last decade, the use of virtual reality (VR) helped clarify the intricate interplay between broader self-related dimensions and dysfunctional eating attitudes and behaviors in women with eating disorders (EDs). The first objective of this study explores the role of body image attitudes (i.e., perceived physical attractiveness, body shape concerns), global self-worth, and physical self-worth in determining visual-perceptual body image representations (i.e., allocentric and egocentric ideal and self-perceived body size) and visual-perceptual body image disturbances (i.e., allocentric and egocentric visual-perceptual body dissatisfaction) in a sample of women with EDs. Additionally, the second objective is to explore the role of body image variables (in terms of attitudes: perceived physical attractiveness, body shape concerns; and in terms of visual-perceptual body image disturbances), global self-worth, physical self-worth, in determining dysfunctional eating attitudes and behaviors (i.e., eating concerns, restraint, and bulimia) in women with EDs.

**Methods:**

The sample involved 96 self-identified female participants. Pearson's bivariate correlations and multiple linear regressions analyses were conducted to investigate the study's objectives. A VR-based figure rating scale was used to perform visual depictive body size estimation tasks in an allocentric and egocentric perspectives.

**Results:**

The findings indicate that physical self-worth and shape concerns are the primary variables related to visual-perceptual body image dissatisfaction. Shape concerns is also associated with eating concerns.

**Discussion:**

This study emphasizes the critical role of physical self-worth and shape concerns as common variables of interest in relation to both visual-perceptual body image representations and disturbances, as well as shape concerns for dysfunctional eating attitudes and behaviors. These findings clarify the understating of the intricate interplay between body image, broader self-related dimensions, and dysfunctional eating attitudes and behaviors in EDs.

## 1 Introduction

Physical appearance plays a significant role in shaping one's identity and sense of self. However, body image is a complex multidimensional phenomenon, including beliefs, emotions, behaviors, and perceptions related to one's body image (Cash, 2004). The current study focuses on the relationship between body image attitudes, self, and physical self related factors in determining visual–perceptual body image representations (VPBRep; self-perceived and ideal body size) and visual–perceptual body image disturbances (VPBDis; the discrepancy between them). Additionally, it explores how these factors relate to dysfunctional eating attitudes and behaviors in women with EDs. Virtual reality (VR) was used as an assessment tool of VPBRep and VPBDis.

Over the past decades, negative body image has been shown to contribute significantly to the continuum of disordered eating attitudes and behaviors (Cash and Pruzinsky, 2002; Monthuy-Blanc et al., 2023a; Stice, 2001, 2002; Turgeon et al., 2015). Within this continuum, various levels and combinations of dysfunctional eating attitudes and behaviors exist, such as restraint, binge-eating episodes, and emotional eating (Monthuy-Blanc et al., 2022c, 2023a,b; Levine and Piran, 2004). At the most severe end of the continuum there are EDs, including anorexia nervosa, bulimia nervosa, and binge-eating disorder, mainly (Monthuy-Blanc et al., 2022c, 2023a,b; Levine and Piran, 2004). Consistent with this, negative body image is a central component in EDs, and overevaluation of the importance of body shape is a transdiagnostic characteristic of EDs (Riva and Dakanalis, 2018). Alongside negative body image, other self-related constructs have been theoretically and empirically connected to EDs psychopathology (Bardone-Cone et al., 2018). For example, the transdiagnostic cognitive-behavioral model of EDs (Fairburn et al., 2003) emphasizes the role of self-esteem as a maintaining mechanism of EDs, while other studies have suggested that low self-esteem may precede and contribute to the development of EDs (Bardone-Cone et al., 2018). However, there is a need for more research to shed light on the role of wider self-concepts in determining dysfunctional eating attitudes and behaviors in individuals struggling with EDs.

**Figure 1:**
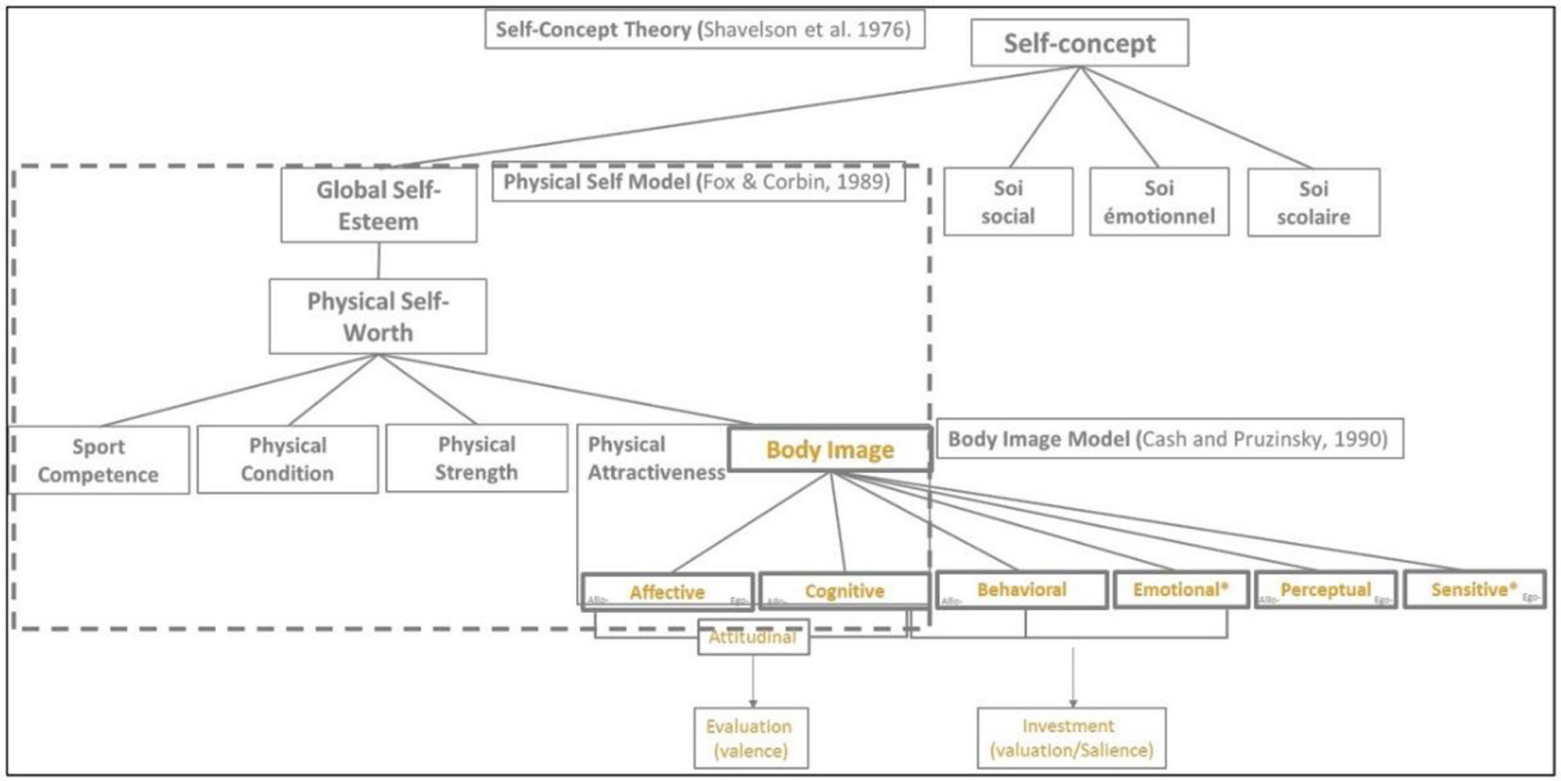
Proposed integration of key conceptual models about self-related concepts and body image. *Dimensions of body image non-theorized in Cash model. Allo, Allocentric representation; Ego, Egocentric representation.

With this question in mind, Fox and Corbin (1989) proposed a multidimensional and hierarchical model that positions physical appearance (i.e., perceived physical attractiveness, which refers to positive body image and the ability to maintain an attractive body over time) as a subdimension of a more comprehensive self-concept (Cash, 2012; Shavelson et al., 1976). Within this framework, physical appearance has been identified as one dimension of several contingencies of self-worth (i.e., academic competence, relationship status, approval from generalized others, and family support; Fox and Corbin, 1989; Shavelson et al., 1976). Global self-worth (i.e., global self-esteem), occupies the upper conceptual level of this model (Brown and Slaughter, 2011). It refers to the overall positive or negative way an individual feel about themselves (Shavelson et al., 1976). The subsequent conceptual level, the domain level, pertains to physical self-worth, which refers to the global feeling of pride, happiness, self-respect, satisfaction, and confidence about one's physical self. Physical self-worth is conceptually further refined, or subdivided, into different perceptions of the physical self, including perceived physical attractiveness (Fox and Corbin, 1989). It has been proposed that the relationships between global self-worth and different perceptions of the physical self, such as physical appearance, among anorexic outpatients reflected simultaneous bottom-up and top-down relationships (Monthuy-Blanc et al., 2012). This implies that a positive or negative shift in global self-esteem or specific sub-domain of physical self-perception influenced the immediate domain or related sub-domains (see Figure 1). However, little is known about the possible contribution of broader self-concepts, such as global self-worth, and physical self-worth, on the determination of one's perceived and ideal body size and shape.

To quote Sarwer et al. (2005) “No discussion of body image assessment is complete without some reference to perceptual aspects of body image” (p. 79). To this regard, together with body image attitudes, Cash (2012) proposes a second dimension of body image, body image perceptions, which refer to an individual's mental representation of their size and shape. Perceptions of body image may result from two processes (McCabe et al., 2006). Firstly, the reception and integration of sensory signals from various sensory inputs (i.e., visual, tactile, and kinesthetic) would allow to perceive the physical dimensions of the body (McCabe et al., 2006; Slade, 1985). Secondly, attitudinal factors, including cognitive and affective factors rooted in an individual's beliefs, experiences, schemas, and prior knowledge about their body, would contribute in shaping their perception of body image (McCabe et al., 2006). In other words, distorted attitudes could influence VPBRep like self-perceived and ideal body size and shape (Cornelissen et al., 2013; Corno et al., 2018; Mölbert et al., 2017a,b). Moreover, the perceived physical attractiveness, introduced by Fox and Corbin model, is also a concept closely related to attitudinal dimension of body image (Cash, 2012). Despite this knowledge and the existing theorical framework, it remains unclear whether variables beyond those strictly associated with body image, such as global and physical self-worth, may explain our body image mental representation.

In terms of VPBRep, research on body image has expanded following the development and implementation of VR-based technologies designed to assess body image and body image disturbances (Ferrer-Garcia et al., 2013; Turbyne et al., 2021). VR is a technology particularly well-suited for understanding body image related phenomena. VR allows to create three-dimensional human bodies that can be personalized and manipulated in terms of, for example, size of different body parts, skin color and body shape. VR technology has been used to overcome some limitations of traditional paper-and-pencil body size estimation tools. Tasks involving body size estimation enable the quantification of VPBDis and body distortion. This is achieved by measuring, respectively, the difference between self-perceived and ideal body sizes, and the difference between actual and self-perceived body sizes (Corno et al., 2022; Ferrer-García and Gutiérrez-Maldonado, 2012). Paper-based figure rating scales, such as the Stunkard Figure Rating Scale (Stunkard et al., 1983), have been widely utilized in visual-perceptual methods to evaluate body dissatisfaction (Doll et al., 2003; Sob et al., 2021). However, they have faced criticism for using figures that illustrate unrealistic depictions of the human body, lacking ecological validity due to their limited reliance on frontal displays (i.e., an allocentric perspective), and for not including figures representing obesity (Doll et al., 2003; Monthuy-Blanc et al., 2022b). Additionally, these figures are two-dimensional, lacking depth and appearing flat. These limitations may hinder participants from identifying with any one figure on the scale (Corno et al., 2024). VR allows to recreate a tri-dimensional and more realistic visual representation of one's perceived, real, and ideal body size and shape. These specific characteristics are thought to facilitate participants' enhanced identification with the three-dimensional bodies (Ferrer-García and Gutiérrez-Maldonado, 2012). Another appeal of VR lies in the ability to view virtual bodies either from an egocentric perspective (i.e., seen from the first-person view, as if being in the body) or from an allocentric perspective (i.e., seen from the third person, as if observing another person or looking at oneself in a mirror). These features allow the person to experience the virtual body as if it was their own body. This phenomenon is called “embodiment” and represents the replacement of the physical body by a virtual body (Gutiérrez-Maldonado et al., 2018). VR was selected because our hypotheses target perspective-sensitive, visual–perceptual indices of body image (VPBRep/VPBDis). By providing 3D, manipulable avatars and both allocentric and egocentric viewpoints, VR allows direct testing of whether self-related variables differentially predict VPBRep/VPBDis across perspectives and how these indices relate to dysfunctional eating behaviors.

Previous VR studies often vary in how these perspectives are implemented and validated (e.g., inconsistent mirror setups, unmeasured or weak embodiment, limited avatar personalization/calibration, and heterogeneous task instructions), which can constrain interpretability and between-study comparison (Ferrer-Garcia et al., 2013; Ferrer-García and Gutiérrez-Maldonado, 2012; Guy et al., 2023). Also, although VR is a promising method for studying the complex interplay between broader self-related dimensions and dysfunctional eating attitudes and behaviors, there is still a limited body of literature specifically addressing this relationship among people with EDs (Riva et al., 2019; Gutiérrez-Maldonado et al., 2016, 2015; Perpiñá et al., 1999). Results from different countries show that the mental representation of the body in an egocentric perspective is different than in an allocentric perspective in clinical sample of women with EDs (Turbyne et al., 2021; Ferrer-García and Gutiérrez-Maldonado, 2012; Riva, 1998). To our knowledge, no empirical study focused on VR to examine the complex interplay between broader self-related dimensions and dysfunctional eating attitudes and behaviors specifically in a sample of women with EDs. Adding knowledge about the role of body image attitudes and physical self (both component often overlooked when studying eating disorders) could help generate new perspective in the treatment of those disorders. Moreover, integrating VR to explore these variables would facilitate not only symptom detection but also help sensibilize to the lived experience of the patients we are trying to help. Even maybe, creating a space where new treatment could be explored.

To address this gap in scientific knowledge, this study had two main objectives. The first objective explores the role of body image attitudes (i.e., perceived physical attractiveness, body shape concerns), global self-worth, and physical self-worth in determining VPBRep (i.e., VR allocentric and egocentric ideal and self-perceived body size) and VPBDis (i.e., VR allocentric and egocentric) in a sample of women with EDs. The second objective seeks to explore the role of body image variables (in terms of attitudes: perceived physical attractiveness, body shape concerns; and in terms of VPBDis: allocentric and egocentric), global self-worth and physical self-worth, in determining dysfunctional eating attitudes and behaviors (i.e., eating concerns, restraint, and bulimia) in women with EDs. Due to the exploratory nature of the objectives, no hypotheses were proposed.

## 2 Material and methods

### 2.1 Design and participants

The sample consisted of 96 self-identified female participants. Male participants were excluded due to their insufficient number (*n* = 11) which would have reduced statistical power and introduce imbalance in our analyses. Age ranged from 18 to 84 years old (*M* = 43.31, *SD* = 14.74), and BMI from 13.49 to 59.30 kg/m^2^ (*M* = 32.90, *SD* = 10.62). Based on DSM-5 criteria, 14 (17.7%) participants received a diagnosis of anorexia nervosa, 16 (20.3%) a diagnosis of bulimia which, 29 (36.7%) a diagnosis of binge eating disorder, 18 (22.8%) unspecified feeding or eating disorder, and 2 (2.5%) other specified feeding and eating disorder. Given the small sample size and the exploratory nature of our objectives, we decided to include all participants despite heterogeneity in age and diagnostic categories, in order to preserve statistical power. While different diagnoses might influence perceptions differently, exploring these subgroup effects was not the focus of the present study. We acknowledge that this heterogeneity might influence the internal validity of our results, therefore interpretations will be made with caution. Participants provided written informed consent and allowed the use of their anonymous data in accordance with current legislation regarding the personal data protection (World Medical Association, 2018). This study obtained the approval from the ethics committee of Université du Québec à Trois-Rivières (Quebec, Canada; reference number: CER-22-293-10.02).

### 2.2 Procedure

This retrospective study analyzed anonymized secondary data. Specifically, the data included in this study were collected during the eligibility assessment prior to the enrollment to an *e*Health transdisciplinary program, delivered at the university clinic, from September 2017 to December 2022 (Monthuy-Blanc et al., 2022a; St-Pierre et al., 2022). Participants with severe comorbid psychiatric conditions (e.g., personality disorders, psychosis or severe anxiety or depression) and those who were unable to understand French were excluded from the program. Eligibility to enroll to the program was evaluated by a transdisciplinary clinical team. The criteria for data extraction included self-identification as a woman, self-reported age of 18 or older, and have confirmed a diagnosis of EDs. Diagnoses of EDs and comorbidities were assessed by the transdisciplinary clinical team of the eLoriCorps Program and confirmed by a diagnostic specialist (physician or psychologist) using DSM−5-TR criteria [(Monthuy-Blanc et al., 2015; St-Pierre et al., 2023) for a detailed description of data source].

### 2.3 Assessment measures

Participants were asked information about self-reported age and current height and weight (to calculate the Body Mass Index, BMI, kg/m^2^).

*eLoriCorps-Immersive Body Rating Scale version 1.1* (*e*LoriCorps-IBRS 1.1; Monthuy-Blanc et al., 2022b). This VR-based figure rating scale was used to perform visual depictive body size estimation tasks in an allocentric and egocentric perspectives (Mölbert et al., 2017b). Participants were asked to select the virtual body that closely represented their ideal and perceived body size. *Z*-scores were first calculated separately due to the use of different scales (i.e., a seven-point and a nine-point scale of *e*LoriCorps-IBRS 1.1). Since both sets of *Z*-scores calculated were on the same standardized scale, they were then merged into a single variable for analysis across all participants, including Z ideal body size—Allocentric, Z Ideal body size Egocentric, Z Perceived body size Egocentric, Z Perceived body size Allocentric. VPBDis refers to the difference between perceived and ideal body size. A score different than 0 indicates dissatisfaction with one's body. A positive score indicates that women's ideal body size was thinner that their perceived body size, while a negative score suggests that participants' ideal body size was bigger than their perceived body size (for a detailed description of the procedure please refer to Monthuy-Blanc et al., 2022b). *Z*-scores were computed for egocentric (i.e., Z VPBDis-Egocentric) and allocentric (i.e., Z VPBDis-Allocentric) visual-perceptual body dissatisfaction.

*Eating Disorder Examination Questionnaire* (EDE-Q; Fairburn and Beglin, 1994). The Shape concerns, eating concerns, and restraint subscales of the EDE-Q were used in order to assess participants' body shape concerns, worries about eating and restrained eating, respectively. Participants rated the items on a rating scale ranging from 0 (“no days”) to 6 (“every day”). Each subscale's total score was obtained by calculating the average of all responses. In the present study, Cronbach's alpha ranged from α = 0.654 to α = 0.806.

*Physical Self-Inventory* (PSI; Maïano et al., 2008). The global self-worth, physical self-worth and perceived physical attractiveness subscales of the PSI were used to evaluate participants' global self-worth, physical self-worth, and perceived physical attractiveness according to the model of Fox and Corbin (Fox and Corbin, 1989; Fox, 2000). Answers are rated on a scale ranging from 1 (“not at all”) to 6 (“Absolutely”). In the present study, Cronbach's alpha ranged from α = 0.397 to α = 0.810. The relatively low values (i.e., α = 0.397) likely come from the fact that each subscale includes only two items, which naturally limits internal consistency estimates, because of Cronbach's alpha sensitivity to the number of items. However, this scale has been widely used and validated for assessing physical self variables supporting our decision to retain it for our study (Cortina, 1993).

*Eating Disorder Inventory-very short version* (EDI-VS; Maiano et al., 2008). The bulimia subscale of the EDI-VS was used to assess the presence of bulimic attitudes and behaviors using a rating scale from 0 (“not at all”) to 5 (“extremely”). In the present study, Cronbach's alpha was α = 0.831.

### 2.4 Statistical analyses

First, Pearson's bivariate correlations and multiple linear regressions analyses were performed to explore the relationship between allocentric and egocentric visual body image perceptions (i.e., perceived, and ideal body size, VPBDis) and various potential predictors (i.e., shape concerns, perceived physical attractiveness, global self-worth, and physical self-worth). To test the second objective, Pearson's bivariate correlations and multiple linear regressions analyses were conducted to explore the relationship between dysfunctional eating attitudes and behaviors (i.e., eating concerns, bulimia and restraint), body-image related predictors (i.e., shape concerns, perceived physical attractiveness, VPBDis-Egocentric and VPBDis-Allocentric), and broader self-related predictors (i.e., global self-worth, physical self-worth). The normal distribution of the models' residuals was analyzed prior to conducting multiple linear regressions (Field, 2018). To ensure the validity of our multiple linear regression analyses, we also assessed multicollinearity using variance inflation factors and found no problem of multicollinearity with our variables. The Breusch–Pagan test indicated heteroscedasticity in only one model (*p* = 0.02) and the same thing was found for residue independence (*p* = 0.044). To address those problems, we report heteroscedasticity-robust standard errors (HC3) for those two models. Missing values were checked for their randomness. Little's test indicated that data were missing completely at random (MCAR; *p* > 0.05; Tabachnick and Fidell, 2007). Listwise deletion was used to handle missing data and statistics were conducted using IBM SPSS, version 27.

## 3 Results

Descriptive statistics for body image variables, global self-worth, physical self-worth, and dysfunctional eating attitudes and behaviors are reported in Table 1. Regarding body image variables, women reported clinically significant levels of body shape concerns. Additionally, when performing the VR-based visual depictive body size estimation task in an allocentric condition, participants ideal body size was on average slightly smaller than their perceived body size. When assessing body sizes in an egocentric condition, women ideal body size was on average slightly bigger than their perceived body size. Women's responses in both scenarios indicated low VPBDis.

**Table 1:** Descriptive statistics for body image and dysfunctional eating attitudes and behaviors.

	***M***	***SD***	**Min–Max**
Perceived body size-Allo-7p	4.69	1.51	2–7
Perceived body size-Ego-7p	4.61	1.40	2–7
Ideal body size-Allo-7p	3.15	0.80	1–4
Ideal body size-Ego-7p	3.22	0.87	1–5
VPBDis-Allo-7p	1.54	1.53	−2–5
VPBDis-Ego-7p	1.39	1.46	−1–4
Perceived body size-Allo-9p	7.05	1.46	4–9
Perceived body size-Ego-9p	6.69	1.44	3–9
Ideal body size-Allo-9p	4.07	1.02	1–7
Ideal body size-Ego-9p	3.88	0.96	1–6
VPBDis-Allo-9p	2.98	1.62	0–8
VPBDis-Ego-9p	2.77	1.49	0–8
Physical attractiveness	2.83	1.03	1.00–5.50
Shape concerns	4.64	1.27	0.50–6.00
Global self-worth	2.06	0.84	1.00–4.50
Physical self-worth	2.41	1.15	1.00–6.00
Restraint	2.79	1.44	0.00–5.80
Bulimia	5.72	2.85	0.00–10.00
Eating concerns	4.12	1.39	0.60–6.00

M, Mean; SD, Standard deviation; Min–Max, Minimum-Maximum score; Perceived body size-Allo-7p, Perceived body size measured in the allocentric perspective on a seven-point Likert scale; Perceived body size-Ego-7p, Perceived body size measured in the egocentric perspective on a seven-point Likert scale; Ideal body size-Allo-7p, Ideal body size measured in the allocentric perspective on a seven-point Likert scale; Ideal body size-Ego-7p, Ideal body size measured in the egocentric perspective on a seven-point Likert scale; VPBDis-Allo-7p, Allocentric visual perceptual body dissatisfaction calculated with the seven-point Likert scale; VPBDis-Ego-7p, Egocentric visual perceptual body dissatisfaction calculate with the seven-point Likert scale; Perceived body size-Allo-9p, Perceived body size measured in the allocentric perspective on a nine-point Likert scale; Perceived body size-Ego-9p, Perceived body size measured in the egocentric perspective on a nine-point Likert scale; Ideal body size-Allo-9p, Ideal body size measured in the allocentric perspective on a nine-point Likert scale; Ideal body size-Ego-9p, Ideal body size measured in the egocentric perspective on a nine-point Likert scale; VPBDis-Allo-9p, Allocentric visual perceptual body dissatisfaction calculated with the nine-point Likert scale; VPBDis-Ego-9p, Egocentric visual perceptual body dissatisfaction calculate with the nine-point Likert scale. Seven-point Likert scales: n = 36; nine-point Likert scales: n = 58.

### 3.1 Predictors of visual-perceptual body image representations and disturbances

Results of bivariate Pearson's correlations between global self-worth, physical self-worth, attitudinal, and visual-perceptual body image variables are reported in Table 2.

**Table 2:** Pearson's bivariate correlations between body image variables as well as broader self- (i.e., global self-worth), and physical self-concepts (i.e., physical self-worth).

	**1**	**2**	**3**	**4**	**5**	**6**	**7**	**8**	**9**	**10**
1. Z Perceived body size—egocentric	–									
2. Z Perceived body size—allocentric	0.490^***^	–								
3. Z Ideal body size—egocentric	0.349^**^	0.153	–							
4. Z Ideal body size—allocentric	−0.027	0.323^**^	0.380^***^	–						
5. Z VPBDis—egocentric	0.787^***^	0.395^***^	−0.302^**^	−0.272^*^	–					
6. Z VPBDis—allocentric	0.508^***^	0.815^***^	−0.066	−0.270^*^	0.555^***^	–				
7. Global self-worth	−0.260^*^	−0.279^*^	0.097	0.073	−0.327^**^	−0.318^**^	–			
8. Physical self-worth	−0.427^***^	−0.538^***^	−0.040	−0.082	−0.402^***^	−0.479^***^	669^***^	–		
9. Physical attractiveness	−0.265^*^	−0.372^***^	0.147	0.007	−0.361^**^	−0.371^***^	0.543^***^	0.551^***^	–	
10. Shape concerns	0.462^***^	0.331^**^	−0.019	−0.137	0.479^***^	0.416^***^	−0.586^***^	−0.436^***^	−0.573^***^	–

Z Perceived body size—egocentric, Z score of perceived body size measured in the egocentric perspective; Z Perceived body size—allocentric, Z score of perceived body size measured in the allocentric perspective; Z Ideal body size—egocentric, Z score of ideal body size measured in the egocentric perspective; Z Ideal body size—allocentric, Z score of ideal body size measured in the allocentric perspective; Z VPBDis—egocentric, Z scores of egocentric visual perceptual body dissatisfaction; Z VPBDis—allocentric, Z score of allocentric visual perceptual body dissatisfaction.

^*^p < 0.05, ^**^p < 0.01, ^***^p < 0.001.

Multiple linear regressions were conducted to predict allocentric and egocentric ideal and perceived body size and VPBDis based on attitudinal body image variables, global self-worth, and physical self-worth (Table 3). A correction was applied to control for the possibility of false discovery rates (FDR) due to multiple testing. The Benjamini–Hochberg correction method was selected given the large number of regressions done here.

**Table 3:** Linear models of predictors of visual-perceptual body image representations and disturbances, as well as dysfunctional eating attitudes and behaviors.

	***b***	***SE B***	**β**	***p***	***P* (corrected)**	***sr* ^2^**
**Z VPBDis—egocentric**						
Constant	−1.544	0.791		0.055	0.171	*0.050*
Global self-worth	0.128	0.162	0.116	0.043	0.659	0.008
Physical self-worth	−0.195	0.112	−0.248	0.086	0.231	0.040
Physical attractiveness	−0.016	0.126	−0.017	0.899	0.919	0.0002
Shape concerns	0.270	0.098	0.370	0.007	0.052	0.095
BMI	0.015	0.009	0.175	0.103	0.246	0.036
**Z VPBDis—allocentric**						
Constant	−1.259	0.800		0.120	0.267	0.032
Global self-worth	0.195	0.162	0.170	0.233	0.441	0.019
Physical self-worth	−0.302	0.114	−0.367	0.009	0.062	0.087
Physical attractiveness	−0.032	0.126	−0.032	0.802	0.871	0.0009
Shape concerns	0.215	0.099	0.284	0.032	0.135	0.060
BMI	0.019	0.009	0.213	0.042	0.135	0.055
**Z perceived body size—egocentric**						
Constant	−2.699	0.760		0.0007	0.010	0.146
Global self-worth	0.275	0.154	0.235	0.078	0.216	0.041
Physical self-worth	−0.280	0.108	−0.334	0.012	0.052	0.083
Physical attractiveness	0.088	0.119	−0.087	0.465	0.531	0.007
Shape concerns	0.338	0.094	0.437	0.006	0.024	0.149
BMI	0.029	0.009	0.318	0.002	0.024	0.129
**Z perceived body size—allocentric**						
Constant	−0.719	0.765		0.350	0.583	0.012
Global self-worth	0.260	0.155	0.226	0.098	0.158	0.037
Physical self-worth	−0.376	0.109	−0.458	0.0009	0.034	0.139
Physical attractiveness	−0.089	0.120	−0.090	0.463	0.510	0.007
Shape concerns	0.103	0.094	0.136	0.277	0.510	0.016
BMI	0.026	0.009	0.292	0.004	0.030	0.106
**Eating concerns**						
Constant	0.454	1.083		0.676	0.789	0.003
Global self-worth	−0.040	0.218	−0.025	0.854	0.907	0.0005
Physical self-worth	−0.085	0.158	−0.073	0.592	0.730	0.004
Physical attractiveness	0.124	0.167	0.087	0.463	0.659	0.008
Shape concerns	0.796	0.138	0.738	0.0000002	0.00001	0.323
Z VPBDis—egocentric	−0.242	0.168	−0.163	0.153	0.326	0.029
Z VPBDis—allocentric	0.060	0.165	0.042	0.718	0.801	0.002
BMI	−0.005	0.013	−0.035	0.723	0.801	0.002
**Restraint**						
Constant	3.623	1.181		0.003	0.026	0.117
Global self-worth	−0.495	0.236	−0.295	0.039	0.135	0.058
Physical self-worth	0.361	0.171	0.299	0.038	0.135	0.059
Physical attractiveness	0.020	0.183	0.014	0.913	0.919	0.0002
Shape concerns	0.310	0.150	0.278	0.042	0.135	0.057
Z VPBDis—egocentric	0.203	0.183	0.133	0.271	0.470	0.017
Z VPBDis—allocentric	0.337	0.180	0.227	0.066	0.198	0.047
BMI	−0.067	0.137	−0.508	0.000006	0.0002	0.252

Z Perceived body size—egocentric, Z score of perceived body size measured in the egocentric perspective; Z Perceived body size—allocentric, Z score of perceived body size measured in the allocentric perspective; Z VPBDis—egocentric, Z scores of egocentric visual perceptual body dissatisfaction; Z VPBDis—allocentric, Z score of allocentric visual perceptual body dissatisfaction.

For egocentric and allocentric ideal body size, the regression models were not statistically significant [egocentric ideal body size: *F*_(5, 73)_ = 1.694 *p* = 0.147, *R*^2^ = 0.043; allocentric ideal body: *F*_(7, 74)_ = 1.074, *p* = 0.382, *R*^2^ = 0.005].

Regarding egocentric perceived body size, the regression equation was statistically significant [*F*_(5, 75)_ = 11.13, *p* < 0.001], with an *R*^2^ of 0.391, meaning that the variables in this regression explained about 39% of the variance in egocentric perceived body size. More specifically, physical self-worth had a negative impact (*t* = −2.591, *p* = 0.012), while shape concerns and BMI had a positive one (respectively, *t* = 0.734, *p* = 0.006 et *t* = 3.308, *p* = 0.002). However, after the FDR correction, only shape concerns and IMC stayed significant (with both *p*-values of 0.024), indicating that those participants with higher shape concerns and IMC were expected to select larger self-perceived body sizes in the egocentric perspective. The regression model for allocentric self-perceived body size, which was statistically significant [*F*_(5, 74)_ = 9.793, *p* < 0.001] with an *R*^2^ of 0.358, revealed a different scenario. Physical self-worth and BMI were the only predictors that had a significant weight, even after the correction. After the correction, physical self-worth had a negative weight (*t* = −3.462, *p* = 0.034) suggesting that women who self-reported higher physical self-worth were expected to select smaller allocentric self-perceived body sizes. On the other hand, BMI had a positive weight (*t* = 2.962, *p* = 0.030) suggesting that women who had higher BMI were expected to select higher allocentric self-perceived body sizes.

Regarding egocentric visual-perceptual body dissatisfaction, the regression equation was statistically significant [*F*_(5, 73)_ = 6.444, *p* < 0.001], with an *R*^2^ of 0.259. As reported in Table 3, shape concerns had a significant positive weight (*t* = 2.77, *p* = 0.007), indicating that those women with hig/her shape concerns were expected to have higher egocentric body dissatisfaction. However, after the correction, this relation remained only marginally significant (*p* = 0.052). Regarding the physical self-worth scale, participants with lower physical self-worth were expected to have higher VPBDis-Egocentric, but this relationship was only marginally significant and disappeared after the correction. Regarding VPBDis-Allocentric, the regression equation was statistically significant [*F*_(5, 74)_ = 7.693, *p* < 0.001], with an *R*^2^ of 0.298. Physical self-worth had a significant negative weight, whereas BMI and shape concerns had a positive one. However, after the correction those relationships did not remain significant, although the weight of physical self-worth remained marginally significant (*p* = 0.062; see Table 3).

### 3.2 Predictors of dysfunctional eating attitudes and behaviors

Results of bivariate Pearson's correlations between global self-worth, physical self-worth, body image variables, and dysfunctional eating attitudes and behaviors are reported in Table 4.

**Table 4:** Pearson's bivariate correlations between body image variables and dysfunctional eating attitudes and behaviors.

	**1**	**2**	**3**	**4**	**5**	**6**	**7**	**8**	**9**
1. Z VPBDis—egocentric	–								
2. Z VPBDis—allocentric	0.551^***^	–							
3. Global self-worth	−0.315^**^	−0.304^**^	–						
4. Physical self-worth	−0.389^**^	−0.483^***^	0.664^***^	–					
5. Physical attractiveness	−0.376^**^	−0.366^**^	0.500^***^	0.552^***^	–				
6. Shape concerns	0.486^***^	0.416^***^	−0.550^***^	−0.390^***^	−0.556^***^	–			
7. Eating concerns	0.206	0.193	−0.349^**^	−0.232^*^	−0.268^*^	0.629^***^	–		
8. Restraint	0.171	0.122	−0.193	0.074	−0.080	0.274^*^	0.356^**^	–	
9. Bulimia	−0.035	0.018	0.049	0.041	−0.082	0.158	0.388^**^	−0.074	–

ZVPBDis—egocentric, Z scores of egocentric visual perceptual body dissatisfaction; ZVPBDis—allocentric, Z score of allocentric visual perceptual body dissatisfaction.

^*^p < 0.05, ^**^p < 0.01, ^***^p < 0.001.

Multiple linear regressions were also conducted to predict dysfunctional eating attitudes and behaviors based on body image variables, global self-worth, and physical self-worth. The regression equation was statistically significant for eating concerns [*F*_(7, 70)_ = 8.724, *p* < 0.001], with an *R*^2^ of 0.413. Consistent with the results in Table 3, shape concerns was the only variable that had a significant positive weight, indicating that women who reported higher shape concerns were expected to have higher eating concerns (*t* = 5.783, *p* < 0.001 and this before and after the correction). Regarding restraint, the regression equation was statistically significant [*F*_(7, 71)_ = 6.647, *p* < 0.001], with an *R*^2^ of 0.336. Specifically, global self-worth and BMI had a significant negative weight, whereas physical self-worth and shape concerns had a significant positive regression weight. However, only BMI stayed significant after the correction (*t* = −4.889, *p* = 0.0002). Finally, for bulimia, the regression model was not found statistically significant [*F*_(7, 65)_ = 1.488, *p* = 0.187], with an *R*^2^ of 0.045.

## 4 Discussion

The current study had two objectives: (a) to explore the role of body image attitudes, self and physical self-related factors in determining VBPR and VBPD; and (b) to explore the role of body image attitudes, VBPD, global self-worth and physical self-worth, in determining dysfunctional eating attitudes and behaviors.

### 4.1 Physical self-worth and shape concerns: key predictors of visual-perceptual body image representations and disturbances

The results highlights the relationship between physical self-worth with allocentric VPBDis and egocentric VPBDis with shape concerns. Although shape concerns and body dissatisfaction are both components of body image, they represent distinct constructs. From a cognitive-behavioral perspective, shape concerns refer to the preoccupation with one's shape and to the central role of shape and its control in determining one's self-worth (Fairburn et al., 2003; Fairburn, 2008). It can be distinguished from body image dissatisfaction, which can be defined as the “subjective” negative evaluation of one's body image (Cash, 2012; Stice and Shaw, 2002). It has been suggested that body dissatisfaction may encompass evaluative and affective dimensions of body image, whereas shape concerns could encompass affective and cognitive dimensions (Allen et al., 2008). Moreover, since body dissatisfaction can be assessed by visual-perceptual methods, it could be hypothesized that visual-perceptual body image dissatisfaction may also encompass a perceptual dimension of body image. The results of this study suggest that higher shape concerns could be associated with higher VPBDis whereas higher physical self-worth may be associated with lower VPBDis, providing preliminary evidence about the relationship between these constructs. A similar pattern is observed for VPBRep, but only for one of the two components that is, perceived body rather than ideal body. Overall, we can argue that VPBRep, as well as VPBDis, could not be related solely to attitudinal body image dimensions, but also with broader factors such as physical self-worth and shape concerns. The results also suggest that allocentric and egocentric VPBDis may be associated with different components of body-image perception. However, those results being only marginally significant, they should be interpreted with caution and replicated with a larger and more robust sample.

### 4.2 Global self-worth, physical self-worth, and shape concerns: key predictors of dysfunctional eating attitudes and behaviors

Regarding the second objective, the variance of eating concerns was significantly associated only with shape concerns, and restraint only with BMI after the correction, whereas the regression model of bulimia was not found statistically significant.

The role of shape concerns in association with eating concerns finds its support in the cognitive-behavioral transdiagnostic approach to EDs. This approach posits shape concerns alongside weight concerns, as the distinctive “core psychopathology” of EDs (Fairburn, 2008). Shape concerns can drive eating concerns by promoting restrictive eating to achieve a certain ideal shape. Dietary restraint, indeed, has been shown to be significantly affected by self-esteem (Kong et al., 2012). Interestingly, neither body dissatisfaction nor shape concerns were significantly associated with restraint after the correction. This could be due to the lack of statistical power, since the sample is small. However, it has been suggested that body dissatisfaction may exert an effect on restrained eating through the mediation of self-esteem (Kong et al., 2012). Future studies are needed to elucidate the relationship between self-esteem, body image variables and restrictive eating. No body image-related, neither self-related variable was significantly related to bulimic attitudes and behaviors, which may also be due to a lack of statistical power. While previous studies have indicated that body dissatisfaction is a risk factor for bulimic pathology (e.g., Stice and Shaw, 2002; Trautmann et al., 2007), there is also evidence that supports the indirect effects of negative body image variables and self-esteem on bulimic attitudes and behaviors. For example, the dual pathway model (Stice, 2001) suggest that body dissatisfaction could be associated to bulimic pathology through either a dietary or negative affect pathway. Another model, known as the three-factor theory proposed by Bardone-Cone et al. (2007), suggests a three-way interaction between high perfectionism, low self-esteem, and high body dissatisfaction as a bulimic pathology model. Overall, to better understand the intricate puzzle that characterizes eating pathology, future research is required.

### 4.3 Strengths and limitations

The current study bears some limitations. Since the sample for this study consisted exclusively of self-identified women, the findings might not be applicable to individuals of different genders. In addition, the recruitment being imbedded in a clinical eligibility assessment, it is possible that a sampling bias affected our sample. Participants with more severe conditions might have been more prone to take part in the clinical program, therefore more likely to be included in this study. Moreover, several measures relied on self-report (e.g., questionnaires and self-selected ratings of perceived and ideal body), which may introduce reporting biases such as social desirability, recall bias, or limited insight, particularly in clinical populations where body image and eating-related cognitions can be distorted. For these reasons and also the small sample of this study the findings of this study should be generalized with caution. Furthermore, the cross-sectional design does not allow to examine the contribution and possible changes of the aforementioned variables over time in determining VPBRep and VPBDis, and problematic eating behaviors. Future studies should include other specific measures regarding dysfunctional eating attitudes and behaviors (e.g., the Bulimic Investigatory Test, Edinburgh, BITE, the Restraint Scale), and also measure other problematic eating attitudes and behaviors (e.g., binge eating, emotional eating; Henderson and Freeman, 1987; Polivy et al., 1988). As the results of this study highlight the importance of self-related variables, it could be interesting to explore in more details different contingencies related to self-worth in determining both VPBRep and VPBDis, as well as dysfunctional eating attitudes and behaviors (Crocker et al., 2003). These findings highlight the importance of considering a broader range of variables when we examine eating disorders. Adopting this perspective may help to develop additional and novel clinical approaches targeting eating disorders attitudes and behaviors. More specifically, in concordance with the previous literature on the subject, VR-based technologies may represent a promising clinical avenue to enhance eating disorders' treatment in general by modifying self-related variables implicated in these conditions (Ferrer-Garcia et al., 2013; Turbyne et al., 2021). However, further research is needed to clarify the underlying mechanisms and determine how such interventions could be implemented effectively.

## 5 Conclusion

The findings of this study have theoretical and practical implications. Theoretically, this study demonstrated the relationship between specific body image variables, broader physical and self-related concepts, and dysfunctional eating attitudes and behaviors in a EDs sample. Practically, the results of this study suggest that intervention and treatment efforts should target shape concerns and physical self-worth. Focusing on these variables, may also help to overcome visual-perceptual body image disturbances and problematic eating behaviors and attitudes.

## Data Availability

The data analyzed in this study is subject to the following licenses/restrictions: The dataset is not publicly available due to privacy and ethical restrictions. Requests to access these datasets should be directed to Research Ethics Boards, comite.ethique@uqtr.ca.
